# Modified ECM-Based Bioink for 3D Printing of Multi-Scale Vascular Networks

**DOI:** 10.3390/gels9100792

**Published:** 2023-10-01

**Authors:** Roni Cohen, Ester-Sapir Baruch, Itai Cabilly, Assaf Shapira, Tal Dvir

**Affiliations:** 1 Department of Biomedical Engineering, Faculty of Engineering, Tel Aviv University, Tel Aviv 6997801, Israel; 2 The Shmunis School of Biomedicine and Cancer Research, Faculty of Life Sciences, Tel Aviv University, Tel Aviv 6997801, Israel; estersapirbaruch@gmail.com (E.-S.B.); itai@cabilly.com (I.C.);; 3 Sagol School of Neuroscience, Tel Aviv University, Tel Aviv 6997801, Israel; 4 Department of Materials Science and Engineering, Faculty of Engineering, Tel Aviv University, Tel Aviv 6997801, Israel; 5 The Sagol Center for Regenerative Biotechnology, Tel Aviv University, Tel Aviv 6997801, Israel

**Keywords:** tissue engineering, vascularization, 3D printing, iPSCs, blood vessels

## Abstract

The survival and function of tissues depend on appropriate vascularization. Blood vessels of the tissues supply oxygen, and nutrients and remove waste and byproducts. Incorporating blood vessels into engineered tissues is essential for overcoming diffusion limitations, improving tissue function, and thus facilitating the fabrication of thick tissues. Here, we present a modified ECM bioink, with enhanced mechanical properties and endothelial cell-specific adhesion motifs, to serve as a building material for 3D printing of a multiscale blood vessel network. The bioink is composed of natural ECM and alginate conjugated with a laminin adhesion molecule motif (YIGSR). The hybrid hydrogel was characterized for its mechanical properties, biochemical content, and ability to interact with endothelial cells. The pristine and modified hydrogels were mixed with induced pluripotent stem cells derived endothelial cells (iPSCs-ECs) and used to print large blood vessels with capillary beds in between.

## 1. Introduction

Vascularization plays a vital role in the survival and function of engineered tissues by supplying them with oxygen and nutrients and removing waste and byproducts. Within the body, oxygen and nutrients diffuse to tissues from surrounding vessels. However, oxygen can only diffuse into tissue at a short distance of 200–300 μm away from a blood vessel. Thus, cells located further than that will not be able to receive oxygen, leading to necrosis and limiting the size of the engineered tissue [1]. The incorporation of blood vessels into engineered tissues is essential for overcoming this limitation by improving oxygenation and nutrient supply to tissues, enhancing tissue function, and facilitating the fabrication of engineered tissues on a large scale [2,3,4].

The vascular system, comprising blood vessels and the heart, transports blood throughout the body [5]. The composition of blood vessel walls varies between vessels of different sizes, as well as between arterial and venous vessels. The larger blood vessels include arteries, veins, arterioles, and venules. These channels are organized into hierarchical networks transporting blood to and from organs and tissues. The wall of a large vessel consists of three layers: the innermost is the tunica intima, which is made of endothelial cells (ECs), a basement membrane (BM), and an internal elastic layer. Enveloping it is a thick layer of smooth muscle named the tunica media. The outermost layer, the tunica adventitia, is a layer of elastic and collagenous fibers [3]. Due to their thick walls and elastic properties, arteries can sustain high pressures of 80–120 mmHg [6]. Contrary to this, a capillary consists of a single layer of ECs surrounded by a BM and a loose covering of pericytes. Throughout the body, capillaries are organized into capillary beds, which are unorganized networks that are dispersed throughout tissues. As a result of their thin walls, they allow for the efficient exchange of oxygen, nutrients, and waste products between the blood and tissues. Individual capillaries are exposed to relatively low pressures of 10–30 mmHg [6].

Considering these differences, when a vascular tissue is engineered, much consideration should be given to the differences in mechanical requirements for each type of blood vessel. As the endothelial cells of the small-scale capillaries are dynamic, they should be engineered within a soft hydrogel. On the contrary, large blood vessels must withstand high blood pressure and flow. Therefore, they should be engineered using stiffer biomaterials [7,8].

Even though blood vessel types differ in their wall composition, EC monolayers cover the lumen of all vessels. These monolayers create a physical and functional barrier at the inner surface of blood vessels, controlling the movement of solutes, cells, and macromolecules between the blood and surrounding tissues [9]. Unique to the EC environment is the specialized extracellular matrix (ECM) commonly referred to as the BM. It covers the endothelium of all blood vessels. Furthermore, it is mainly composed of structural and adhesion proteins. These proteins serve as an anchoring site for cells, a physical barrier, and a signaling hub [10,11]. The structural stability of this microenvironment is mainly provided by collagen IV, while laminin, through its adhesion motifs (such as the short peptide YIGSR), provides the principal cellular binding platform [12]. Mechanical measurements found that Young’s modulus of the adult BM is 1000-fold higher than that of the overlying endothelial layer. This should also be taken into consideration when choosing the biomaterials for vascularization [13].

Several techniques can be used to vascularize engineered tissues, including in vivo vascularization, spontaneous in vitro vascularization, and advanced fabrication techniques [14]. In vivo vascularization relies on the natural ingrowth of blood vessels from the host into the tissue construct upon implantation. Several studies have shown that integrating angiogenic factors into scaffolds populated with cells resulted in functional, in vivo vascularization of the graft [15,16]. It is important to note, however, that the initial thickness of the patch and the cell density within are limited since network formation and anastomosis may take several days. Differently, the spontaneous in vitro vascularization approach relies on the natural ability of ECs to self-assemble into capillary-like structures. Recently, multi-culture systems containing parenchymal and ECs, co-seeded on a scaffold, were used to engineer pre-vascularized tissues [17,18,19,20,21]. Despite their feasibility, the two mentioned approaches resulted in the formation of a random vascular network without the possibility of controlling the location of the vessels, which, when used on their own, could lead to non-vascularized areas in the engineered tissue, causing inefficient perfusion and eventually cell death and necrosis.

The use of advanced fabrication techniques, such as micropatterning and three-dimensional (3D) bioprinting, for tissue vascularization allows us to overcome these challenges by pre-integrating perfusable vascular networks into tissues. Using multi-material 3D bioprinting, vessel architecture could be precisely controlled, and complex vascular networks were created [22,23]. In bioprinting, vascular network architectures are often created with fugitive or sacrificial materials, such as carbohydrate-based glass, Pluronic^®^ F127, and gelatin [24]. By using this method, once the fabrication process is complete, the bulk structure is crosslinked while the sacrificial material is dissolved and evacuated, generating open vascular networks. In this manner, channel networks with specific geometries were created in bulk hydrogel and later seeded with ECs [25,26,27], or bioinks containing ECs were directly printed [28,29,30,31,32]. Later, the channels were perfused with medium to maintain cell viability. The vascularized structures were then anastomosed directly to the host’s vasculature upon implantation, preventing tissue damage and necrosis in the early stages after implantation [33,34].

Recently, our group developed an omentum ECM-based thermoresponsive hydrogel that displays weak mechanical properties at room temperature and physically crosslinks under physiological conditions [35]. Later, this hydrogel was used as a bioink to 3D print both the parenchymal tissue and the blood vessels in cardiac patches and whole hearts [28].

**Figure 1:**
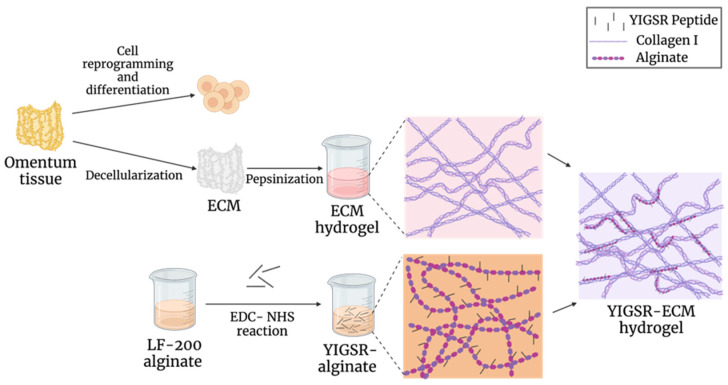
Schematics of the hybrid hydrogel generation. ECM hydrogel was made from omental tissue, which can be easily extracted from patients. Cells and ECM were then separated. Pluripotent stem cells were induced, while the ECM was processed into a thermoresponsive hydrogel. Using carbodiimide activation, YIGSR peptides were conjugated to alginate, which was integrated with the ECM hydrogel to create an ECM-based hydrogel with enhanced mechanical properties and endothelial cell-specific adhesion motifs.

Here, we sought to develop a modified ECM bioink, with enhanced mechanical properties and endothelial cell-specific adhesion motifs, to serve as a building material for 3D printing multiscale blood vessel network. The modified hydrogel was composed of a combination of natural ECM and alginate conjugated with a laminin adhesion molecule motif Tyr-Ile-Gly-Ser-Arg (YIGSR) (Figure 1). This peptide interacts with integrins to anchor cells to the ECM, thus regulating cell behavior and facilitating cell adhesion, proliferation, and migration [36]. Following this, the hybrid hydrogel was characterized to evaluate its mechanical properties, biochemical content, and its ability to interact with endothelial cells. The pristine and modified ECM-based hydrogels were co-printed to generate large blood vessels with capillary beds in between.

## 2. Results and Discussion

### 2.1. Fabrication of the YIGSR-ECM Hydrogel

**Figure 2:**
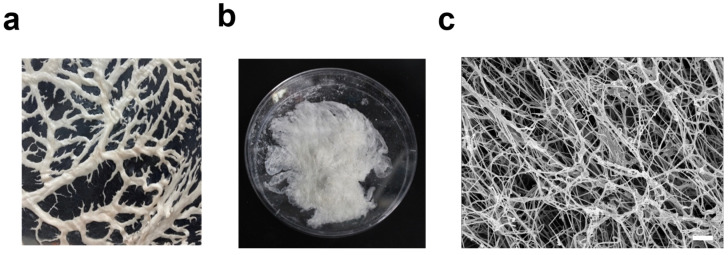
Omentum decellularization and ECM hydrogel generation. (**a**) Native omentum. (**b**) Decellularized omentum. (**c**) Representative hrSEM image of the hydrogel. Scale bar = 1 μm.

In order to generate the hybrid bioink, native porcine omental tissue (Figure 2a) was decellularized by physical, chemical, and enzymatic processes as previously described [35,37]. The decellularized tissue (Figure 2b) was ground and pepsinized to produce an ECM hydrogel. High-resolution scanning electron microscopy (hrSEM) examination of the ECM hydrogel revealed fibrous nanostructures with an average fiber diameter of 55 ± 0.004 nm (Figure 2c).

Next, the YIGSR-alginate conjugate was synthesized by linking the functional peptide with the alginate backbone via an amide bond. NH_2_-GGGGYIGSRGGGG-Me was selected as the specific peptide sequence for modification. On both sides of the sequence, glycine spacers were added to improve chain flexibility and peptide–receptor interaction [38,39]. Additionally, the peptide was designed so that an amine group appears only on one side of the sequence, preventing double conjugation with alginate. High G content, high-viscosity alginate (LF200, FMC BioPolymer) alginate was chosen as the specific type of alginate due to its long polysaccharide chains and enhanced mechanical properties compared to other alginate types.

**Figure 3:**
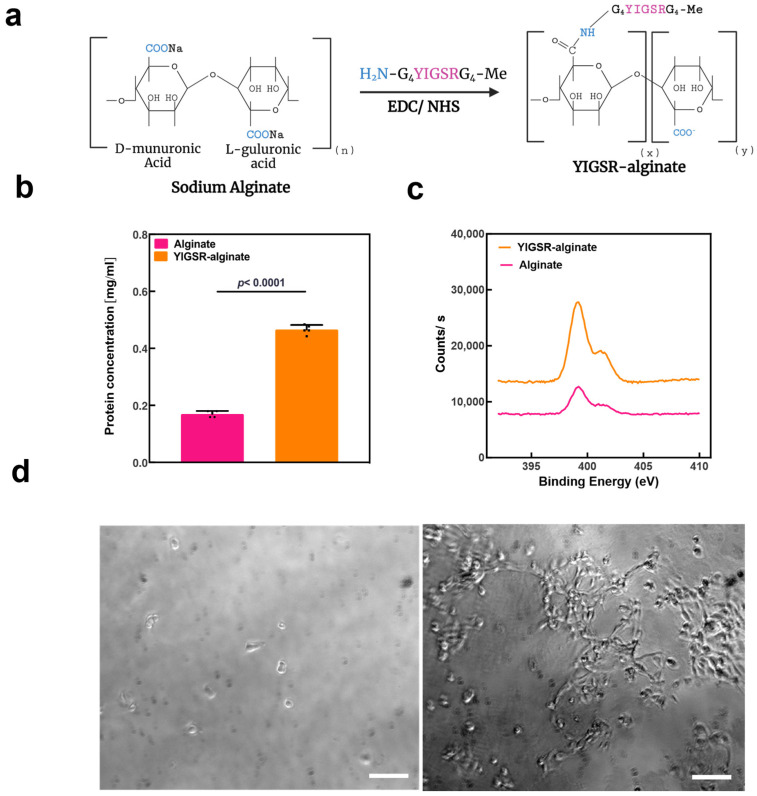
Alginate modification with the biologically active YIGSR peptide. (**a**) YIGSR-alginate gel generation schematics. (**b**) YIGSR-alginate characterization by spectrophotometry. Protein content in LF200 samples (pink) and YIGSR-alginate (orange) (*n* = 3). (**c**) YIGSR-alginate characterization by XPS. Representative N1s scans of the alginate sample (pink) and the YIGSR-alginate sample (orange). (**d**) Adhesion of iPSCs-derived ECs to pristine (**left**) and modified alginate (**right**) sheets, 4 h post-seeding (scale bar = 100 μm).

The modification of alginate was achieved via carbodiimide chemistry with 1-ethyl-3-(3-dimethylamino propyl) carbodiimide (EDC) and N-hydroxysulfosuccinimide (Sulfo-NHS) coupling agents [40,41,42]. The modification resulted in amide bonds between the alginate carboxylic groups and amines on the peptide sequence. The schematic synthesis steps of YIGSR-alginate are shown in Figure 3a.

The successful amidation of the alginate with YIGSR peptide was evaluated by spectrophotometry and X-ray photoelectron spectroscopy (XPS). Spectrophotometry was used to measure absorbance at 280 nm in unmodified alginate samples and YIGSR-alginate samples. The comparison showed a significant increase in absorption at 280 nm in the modified alginate samples, which can be attributed to the increase in aromaticity due to the introduction of tyrosine into the alginate. This proved the success of the modification and allowed protein concentration quantification using the Beer–Lambert law (Figure 3b). Additionally, when the samples were analyzed using XPS, it was shown that the successful amidation of alginate led to a rise in the N1s XPS signals to approximately 1.5 atomic % (Figure 3c). This stems from the established amide bonds between the alginate and the peptide, as well as from the amide groups of the YIGSR peptide.

Upon fabrication of YIGSR-alginate, a 2D cell seeding assay was performed to evaluate the biological activity of the material. ECs derived from iPSCs were seeded on calcium crosslinked unmodified and modified alginate sheets. Assessment of cell adhesion and morphology was performed four hours after seeding. Unmodified alginate sheets showed round and scarce cells. Contrary, YIGSR-alginate sheets had a high number of attached cells that appeared elongated and spread (Figure 3d).

Next, the hybrid hydrogels were prepared by mixing YIGSR-alginate solution with ECM hydrogel at a 1:2 volume ratio, resulting in 0.6% YIGSR-alginate and 1% ECM concentrations (*w*/*v*) in the final product. This ratio was chosen to provide an effective ECM concentration for the hydrogel’s biological activity while maximizing alginate content to improve its mechanical properties. Alginate concentrations in the hybrid hydrogel and in the YIGSR-alginate solution were limited by the solubility of the modified product. The hydrogels were then crosslinked, as discussed in the next section.

### 2.2. Characterization of the YIGSR-ECM Hydrogel

**Figure 4:**
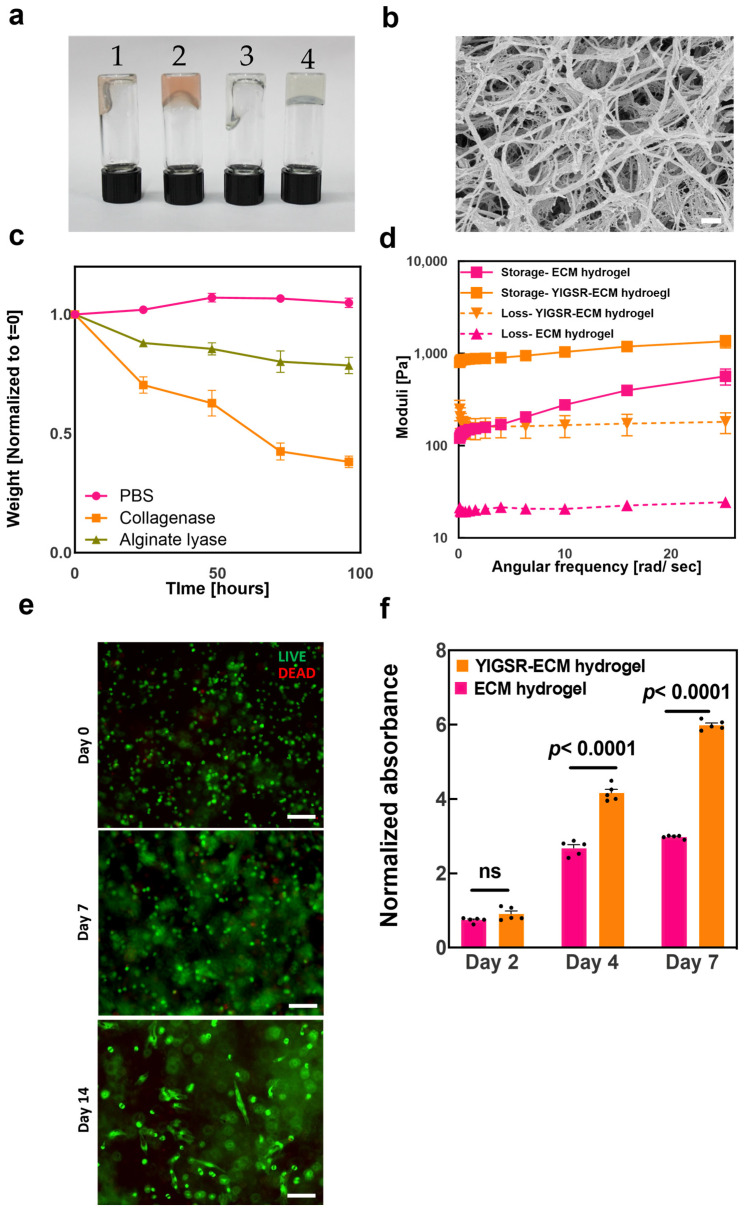
Enhancing the mechanical properties and biological activity of the ECM hydrogel by integration with the YIGSR-alginate gel. (**a**) ECM hydrogel at room temperature (1) and after gelation at 37 °C (2). YIGSR-ECM Hydrogel at room temperature (3) and after ionic crosslinking and gelation at 37 °C (4). (**b**) Representative hrSEM image of a sample of the YIGSR-ECM hydrogel crosslinked with 25 mM calcium D-gluconate (scale bar = 400 nm). Rheology measurements of crosslinked 1% ECM (pink) and YIGSR-ECM hydrogel crosslinked with 25 mM calcium D-gluconate (orange) upon frequency sweep. (**c**) YIGSR-ECM hydrogel swelling and degradation. Swelling and degradation profiles under hydrolytic conditions compared to enzymatic treatment with collagenase and alginate lyase (*n* = 4). (**d**) Storage modulus (continuous lines) and loss modulus (dashed lines) (*n* = 4). (**e**) Representative microscopy images of LIVE/DEAD staining of HUVECs encapsulated in the modified hydrogel (live cells are stained in green, dead cells are stained in red) (scale bar = 100 μm). (**f**) Quantitative viability assay of HUVECs encapsulated in hydrogels according to PrestoBlue™ assay. Viability is normalized to day 0 (*n* = 5).

We have previously demonstrated that omental ECM hydrogel exhibits weak mechanical properties at room temperature and is physically crosslinked under physiological conditions [35]. As a hybrid, the YIGSR-ECM hydrogel maintains the same properties and can also be further crosslinked by calcium ions to further enhance its mechanical properties (Figure 4a).

HrSEM examination of the YIGSR-ECM hydrogel showed that the overall fibrous microstructures of the ECM hydrogel were maintained in the ionically and thermally crosslinked hybrid hydrogel. However, the microstructure showed a more entangled organization of the fibers with a polymeric mesh in-between them (Figure 4b). This microstructure enables cells to migrate, proliferate, and differentiate, and can also facilitate the diffusion of oxygen, ions, and metabolites between fibers [43,44,45].

YIGSR-ECM hydrogel swelling and degradation were further investigated under hydrolytic and enzymatic conditions. As collagenase, a protease secreted by cells can degrade the native ECM component of our hybrid hydrogel, we assessed the change in hydrogel weight in the presence and absence of the enzyme. Furthermore, alginate lyase, an enzyme that breaks down alginate or alginic acid into smaller molecules, was used as a positive control. As shown in Figure 4c, collagenase treatment for 96 h reduced hydrogel weight by approximately 2.6 fold. It was also found that incubation with PBS did not reduce hydrogel weight but rather slightly increased it, suggesting swelling. The significant weight loss caused by incubation with collagenase demonstrates that the hybrid hydrogel is degradable inside the body. By degrading 3D cell cultures, matrix metalloproteinases facilitate matrix remodeling, cell expansion, and migration within hydrogel networks, thus supporting cell culture inside the hydrogel [46]. Incubation with alginate lyase for 96 h reduced the weight ratio in hydrogels only 1.3 fold compared to untreated samples, which is most likely due to hydrogels’ lower alginate content than ECM content. Although alginate lyase does not exist in the human body, using it to degrade hybrid hydrogels could be beneficial for biomedical encapsulation purposes, such as drug delivery and controlled release. Overall, the reduction in hydrogel weights matches the weight ratio of 2:1 ECM to alginate in the hybrid YIGSR-ECM hydrogel.

The rheological properties of YIGSR-ECM hydrogels were then investigated. In general, a bioink should present the rheological properties associated with viscoelastic hydrogels for 3D bioprinting and blood vessel fabrication specifically [47]. Hybrid hydrogels were compared to the pristine ECM hydrogel. The storage modulus (G′) of the YIGSR-ECM hydrogels was increased by an order of magnitude compared to the storage modulus of ECM hydrogels at the crosslinked state. A similar difference was observed in the loss modulus (G″) (Figure 4d). These enhanced rheological properties better represent the mechanical properties of the endothelial monolayers, basement membranes, and surrounding parenchymal tissue of native blood vessels [13].

We next sought to assess the viability and proliferation of human umbilical vein endothelial cells (HUVECs) in thermally and ionically crosslinked YIGSR-ECM hybrid hydrogels. A live/dead cell assay demonstrated that the hybrid bioink was able to maintain cell viability over two weeks. After 14 days, the cells also began to elongate and formed interconnections with neighboring cells (Figure 4e). In order to further assess cell proliferation inside the hybrid hydrogel, a PrestoBlue metabolic activity assay was used. In both the pristine and modified hydrogels, the population of HUVECs expanded over the course of a week. However, cell proliferation in the modified hydrogel was significantly higher (Figure 4f), probably due to its key role in the interaction of laminin with integrins, regulating cell behavior and facilitating cell adhesion, proliferation, and migration [36].

### 2.3. The 2D and 3D Culture of iPSC-ECs

Immunological rejection is one of the major limitations of cell sources for tissue engineering. Therefore, the use of autologous cells is preferred, in order to eliminate the immunological effects of cellular components [48,49]. iPSCs differentiation into desired cell types allows them to be used for disease modeling [50,51], drug discovery [52,53], drug screening [54] and personalized medicine [55]. Here, we sought to exploit this technology to generate human ECs.

**Figure 5:**
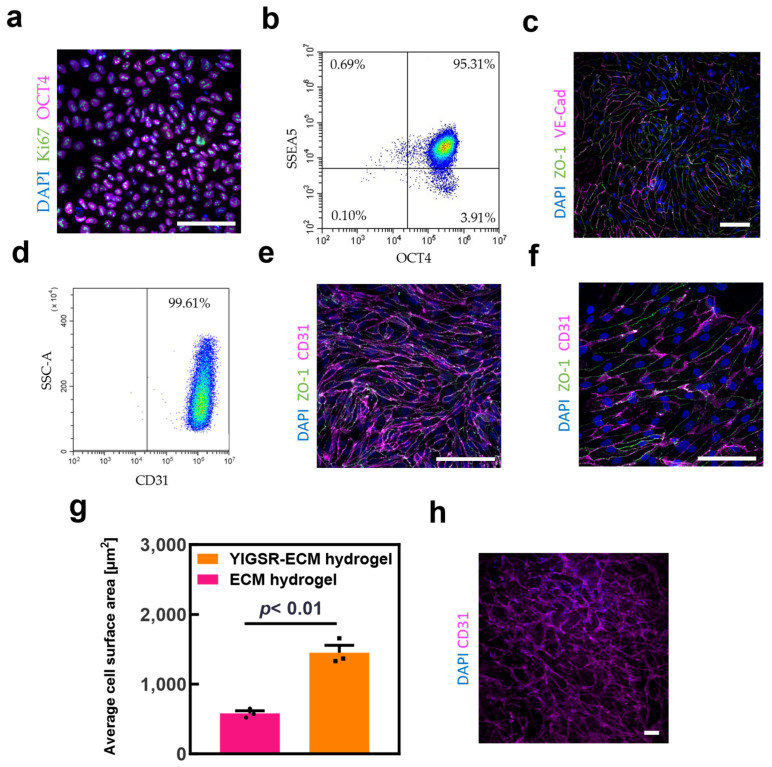
The 2D and 3D culture of iPSC-ECs. (**a**) Representative microscope image of iPSCs immunostained for key pluripotent stem cell markers (scale bar = 100 μm) (pink: Oct4, green: Ki67, and blue: nuclei). (**b**) Flow cytometry analysis of iPSCs for pluripotent stem cells markers SSEA-5 and Oct4. (**c**) Representative microscope image of iPSC-ECs immunostained for key endothelial cell markers (scale bar = 100 μm) (pink: VE-Cadherin, green: ZO-1 and blue: nuclei). (**d**) Flow cytometry analysis of iPSCs for endothelial cells marker CD31 after sorting. iPSC-ECs on ECM hydrogel (**e**), and YIGSR-ECM hydrogel (**f**), immunostained for endothelial cell markers after 2 days in culture (scale bar = 100 μm) (pink: CD31, green: ZO-1 and blue: nuclei). (**g**) Cell surface area quantification using nucleus count on ImageJ (*n* = 3). (**h**) iPSC-ECs encapsulated inside ECM hydrogel immunostained for CD31 after 14 days in culture (scale bar = 100 μm).

Human stromal cells from omental tissues were reprogrammed to become iPSCs as previously described [56]. Immunostaining showed the expression of the pluripotency marker octamer-binding transcription factor 4 (Oct4) and the proliferation marker Ki67, indicating their pluripotent stem cell state (Figure 5a). Immunostaining results were supported by flow cytometry analysis, revealing that over 95% of the iPSCs population expressed Oct4 and the pluripotency marker stage-specific embryonic antigen-5 (SSEA-5) (Figure 5b).

The differentiation of iPSCs into ECs included two main steps of mesoderm induction and endothelial cell induction, mimicking the embryonic development of endothelial cells [57]. At the end of the differentiation, the population was enriched by a magnetic-assisted cell sorting (MACS) process based on the positive endothelial markers vascular-endothelial cadherin (VE-Cad) and CD31. Post MACS, cells were immunostained for the adherence junction VE-Cad and tight junction protein zonna-ocludens 1 (ZO-1), which showed the organization of the cells into an endothelial monolayer with characteristic cobblestone phenotype (Figure 5c) [58,59]. Immunostaining was further confirmed by flow cytometry analysis showing the efficiency of the MACS process as over 99% of the cells expressed the endothelial marker CD31 (Figure 5d).

Following the generation and characterization of iPSC-derived ECs, we sought to investigate their interaction with our hydrogels. While the YIGSR-ECM hydrogel biofunctionality assays demonstrated that it was able to support HUVEC viability and proliferation, no robust 3D organization of the cells was observed. In light of that, and the characteristic monolayer organization of ECs in the lumens of larger blood vessels, we examined their monolayer formation behavior after seeding onto our hydrogels. In a 2D cell seeding assay, iPSC-ECs seeded on the pristine ECM hydrogel (Figure 5e) and hybrid YIGSR-ECM hydrogel (Figure 5f) were analyzed for cell morphology and endothelial cell marker expression. The cells formed monolayers on both hydrogels with the characteristic cobblestone phenotype and a robust expression of CD31 and ZO-1. Interestingly, cells cultured on YIGSR-ECM hydrogels were more spread and had a larger cell surface area (Figure 5g). This may be attributed both to the enhanced mechanical properties and the adhesion motifs [60].

Our goal was to 3D print large blood vessels within the modified hydrogel and small-scale blood vessels within the pristine hydrogel. Therefore, we next investigated the ability of the pristine ECM hydrogel to promote self-assembly of small capillary-like structures. Thus, iPSC-ECs were encapsulated inside the pristine hydrogels and cultured for 14 days. As shown, a network of thin (~10–30 µm) capillary-like vessels was formed inside the hydrogel (Figure 5h).

### 2.4. The 3D Bioprinting of Vascularized Patches

**Figure 6:**
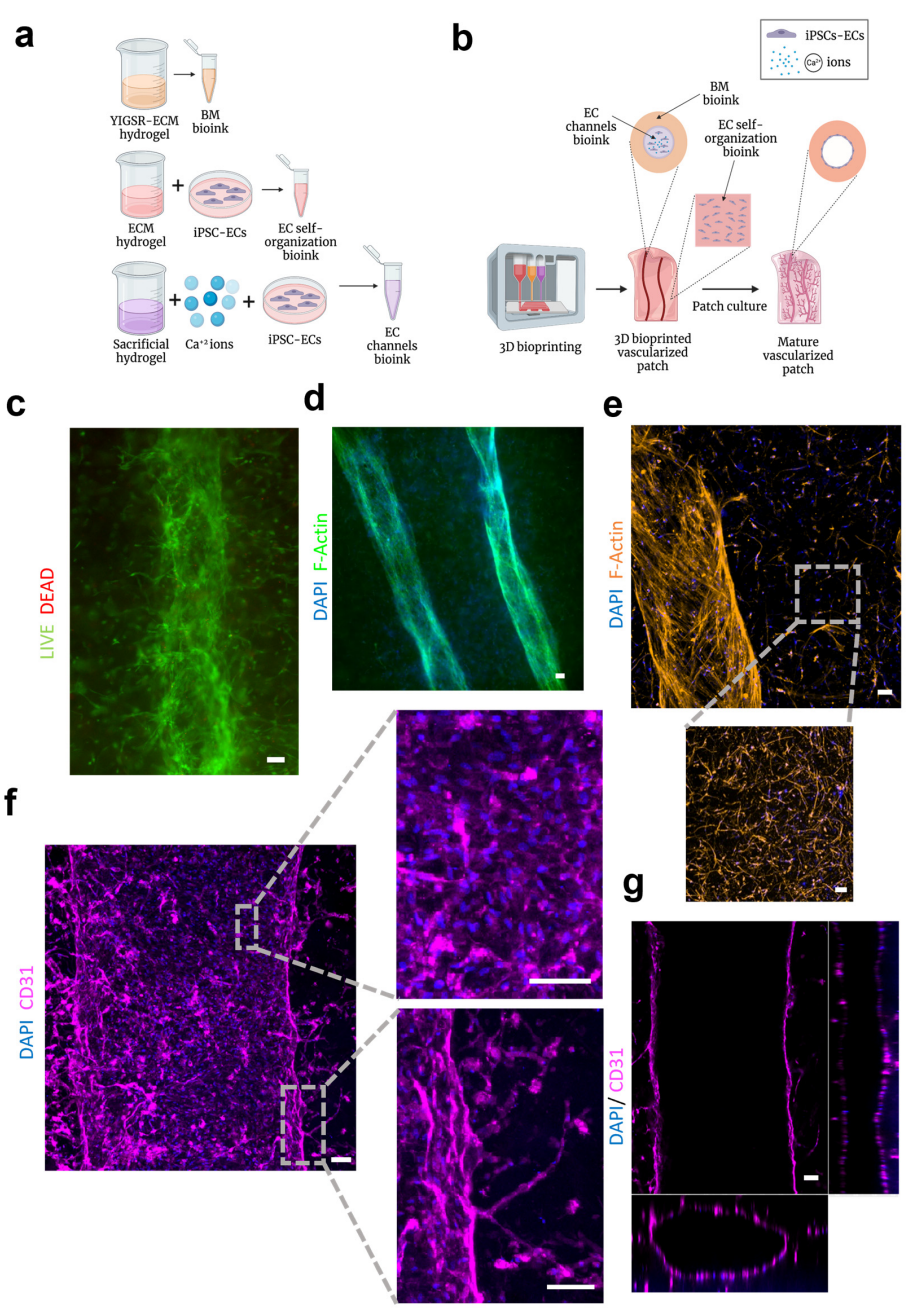
The 3D printed vascularized patches. (**a**) The bioinks that were used in the printing process. (**b**) Schematics of the patch bioprinting process. (**c**) Viability after printing. Representative microscopy images of LIVE/DEAD staining of 3D printed channels 10 days post printing (live cells are stained in green, dead cells are stained in red) (scale bar = 100 μm). (**d**) Representative fluorescence image of two printed large blood vessels stained for F-actin and DAPI after 14 days in culture (scale bar = 100 μm). (**e**) Representative fluorescence image of a printed large blood vessel and surrounding capillary-like structures stained for F-actin and DAPI after 14 days in culture (scale bar = 100 μm). (**f**) Representative 3D confocal images of a 3D printed blood vessel immunostained for the endothelial cell marker CD31 after 14 days in culture, EC monolayer zoom-in image (**top**), a zoom-in image showing EC sprouting from the blood vessel wall (**bottom**) (scale bars = 100 μm). (**g**) Representative 3D confocal images of a 3D printed blood vessel immunostained for CD31 after 14 days in culture (scale bar = 100 μm). Cross-section images, XY plane (**top**), YZ plane (**right**), XZ plane (**bottom**).

We next developed a one-step 3D bioprinting approach for fabricating both large blood vessels with distinct BMs and EC monolayers surrounded by networks of capillary beds. This approach combines three bioinks, including a BM bioink based on the YIGSR-ECM hydrogel, an EC self-assembly bioink comprised of iPSCs-derived ECs mixed with the pristine ECM hydrogel and an EC channels bioink comprised of iPSCs-derived ECs mixed with a sacrificial material and supplemented with calcium ions (Figure 6a,b). First, several layers of the EC self-assembly bioink was deposited to create the capillary beds in the bulk of the patches. On top of that, the BM bioink without cells was deposited in the desired large blood vessel network geometry. Next, the EC channels bioink was deposited inside the BM bioink using a smaller needle gauge. This way, the BM bioink forms the shell of the printed blood vessels, while the EC channels sacrificial bioink forms the core, and ionically crosslinks the BM bioinks due to the calcium ions within. To end the printing process, additional layers of the EC self-assembly bioink were deposited on top of the blood vessel network to create the upper part of the capillary bed. Upon incubation at 37 °C, the EC self-assembly bioink and the BM bioink were thermally crosslinked while the sacrificial EC channels bioink was dissolved, generating a hollow channel network. The ECs in the sacrificial bioink adhered to the channel walls, forming a dense monolayer on the BM hydrogel inner surface. Moreover, the calcium ions in this bioink further crosslinked the BM bioink to enhance its mechanical properties. Additional cultivation of the printed patch promoted EC self-assembly into interconnected capillary-like structures.

First, the biocompatibility of this bioprinting approach was evaluated to ensure that the encapsulation of the cells inside the calcium-loaded sacrificial bioink did not affect cell viability. A LIVE/DEAD assay showed high cell viability 10 days post-printing (Figure 6c). Furthermore, the cells elongated inside the 3D-printed patch, sprouting out of the main blood vessel, further confirming cytocompatibility.

By using printing to fabricate large blood vessels, a variety of geometries can be created. As an example, two large channels were printed in parallel (Figure 6d). This printing configuration allows for the investigation of interactions between neighboring blood vessels. After fabrication, the patches were cultured for two weeks to allow endothelial cell self-assembly in the bulk of the patch, and a monolayer formation on the inner surface of the lumens. After 14 days, the matured patches contained a multi-scale vascular network composed of large blood vessels, with an average diameter of 700 μm, surrounded by a network of thin capillary-like structures, with a diameter of 30 μm (Figure 6e). Printing using extrusion-based techniques cannot produce such small-diameter capillary-like networks because of printing resolution and cell shear stress limitations [61,62,63]. Therefore, both distinct EC bioinks must be incorporated into the same patch to achieve this multi-scale network.

Mature patches were also immunostained for CD31 to detect cell organization. The iPSCs-derived ECs formed a confluent monolayer over the lumen of the printed blood vessels with cells tightly connected. Additionally, EC sprouted from the walls of the larger blood vessels, indicating EC monolayer functionality (Figure 6f).

Shape fidelity is another important aspect of engineered blood vessels. When weak biomaterials are used to fabricate channels, the resulting structures might not be homogeneous and collapse over time. Our printed blood vessels showed high shape fidelity, with round and stable blood vessel lumens (Figure 6g). This is a significant improvement over our previous work, which only utilized the soft ECM hydrogel to fabricate channels [28,32]. This can be attributed to the enhanced mechanical properties of the ionically crosslinked hybrid hydrogel. The fast-crosslinking kinetics of the calcium in the sacrificial bioink allows for the immediate crosslinking of the alginate component in the blood vessel walls, helping to stabilize their structure. In contrast, the ECM component of the patch is only crosslinked when the structures are incubated [64]. This dynamic allows for the creation of robust blood vessels and is critical for their perfusion, as well as for the ability to test blood vessel function assays and allow culturing under flow.

## 3. Conclusions

In the presented work, we have shown the development of an ECM-based hydrogel with improved mechanical properties and EC-specific adhesion motifs. Using this YIGSR-ECM hydrogel and the pristine ECM hydrogel, we were able to 3D print a multi-scale blood vessel network comprised of large cellularized blood vessels and thin capillary beds in between. The YIGSR-ECM bioink-fabricated blood vessels showed good structural fidelity and supported the formation of iPSCs-derived ECs monolayers on the inner surface of its channel.

Other future experiments should examine the integration of these multiscale blood vessel networks with surrounding parenchymal tissue. It is likely that this integration will affect the self-assembly of the ECs in the bulk of the patch. Cell concentrations and culture periods for patch maturation may need to be adjusted to address this issue.

Finally, we believe that this hydrogel offers a great deal of versatility in terms of its use. The alginate component of the hydrogel can be customized by adding different functional peptides. For example, a more general peptide, such as RGD, could be incorporated in place of the more specific YIGSR. Moreover, the mechanical properties of the hydrogel can be tailored to specific applications by selecting different alginate with different properties, changing the concentration of the ionic crosslinker, using a different crosslinker, or altering the ionic crosslinking duration. This will enable us to engineer tissues with different mechanical properties [43].

## 4. Materials and Methods

### 4.1. Materials

Omental tissue from adult pigs was purchased from the institute of animal research in Kibutz Lahav, Israel. Trypsin–EDTA, triton-X100, porcine pepsin (3200–4500 units/mg protein), MES buffer, N-hydroxysulfosuccinimide (Sulfo-NHS), calcium D-gluconate, hoechst 33258, mouse anti-CD-31 antibody (P8590), alginate lyase (10,000 units/mg protein), fluorescein diacetate and propidium iodide were purchased from Sigma-Aldrich (Rheovot, Israel). Primary HUVEC cells and EGM BulletKit Medium were obtained from Lonza (Basel, Switzerland). Fetal bovine serum (FBS), Penicillin/Streptomycin and NutriStem^TM^ were purchased from Biological Industries (Beit Haemek, Israel). Ethyl-3-[3-dimethylaminopropyl]carbodiimide hydrochloride (EDAC) and PrestoBlue™ reagent were purchased from Thermo Fisher Scientific (Kiryat Shmona, Israel). Sodium alginate (Protanal LF200) was purchased from FMC BioPolymer (Sandvika, Norway). NH_2_-GGGGYIGSRGGGG-Me peptides were obtained from GeneScript (Singapore). iPSCs generated from omental stromal cells and were a kind gift from Dr. Rivka Ofir (Ben Gurion University, Beer Sheva, Israel). Matrigel^TM^ and mouse anti-CD144 antibody (555661) were purchased from BD biosciences (Franklin Lakes, NJ, USA). DMEM/F12 and L-Glu were purchased from Sartorius (Beit Haemek, Israel). ReLeSR and Accutase were obtained from STEMCELL technologies. Y-27632 ROCKi and CHIR99021 were purchased from Tocris (Bristol, UK). Neurobasal, N2 and B27 minus retinoic acid were purchased from Gibco (Thermo Fisher Scientific, Kiryat Shmona, Israel). BMP4, VEGF165, forskolin and SB431542 were obtained from PeproTech (Rehovot, Israel). Rabbit anti-ZO1 antibody (CST-13663S) was purchased from Cell signaling. StemPro-34 SFM medium was purchased from Life Technologies (Thermo Fisher Scientific, Kiryat Shmona, Israel). Flow cytometry antibodies SSEA-5 (130-106-716) and control antibody (IS5-21F5), Oct-4 (REA622) and REA control antibody (REA293), CD31 (REA730) and REA control antibody (REA293) were all purchased from Miltenyi Biotech (San Jose, CA, USA). Flow cytometry staining buffer was purchased from R&D systems (Minneapolis, MN, USA). Mouse anti-Oct3/4 antibody (SC-5279) was obtained from Santa Cruz (Santa Cruz, TX, USA). Rabbit anti-Ki67 (ab16667), goat anti-rabbit Alexa 488 (ab150077), goat anti-mouse Alexa 647 (ab150119), goat anti-mouse Alexa 555 (ab150118) antibodies, Phalloidin conjugated to iFluor 647 (ab176759) and phalloidin conjugated to iFluor 555 (ab176756) were purchased from Abcam (Cambridge, UK). Xanthan gum (XG) (XANTURAL 180) was purchased from CP Kelco (San Diego, CA, USA). Collagenase type 2 was purchased from Worthington Biochemical (Lakewood, NJ, USA).

### 4.2. Methods

#### 4.2.1. ECM Hydrogel Production

Previously, our lab has shown the ability of omentum ECM hydrogel to provide a supportive environment to different types of cells [35,37,65]. The omentum ECM hydrogel was produced as described before [39] and kept at 4 °C as a liquid solution until printing. Briefly, omental tissue from adult pigs was agitated for 1 h in hypotonic buffer (10 mM Tris-HCl, 5 mM ethylenediaminetetraacetic acid (EDTA) and 1 μM phenylmethanesulfonyl-fluoride at pH 8.0. The tissue was then subjected to three cycles of freezing (−80 °C) and thawing (37 °C) using the same buffer. After the last cycle, the tissue was gradually dehydrated by washing it once with 70% ethanol for 30 min and three times in 100% ethanol for 30 min each. Polar lipids of the tissue were then extracted by three 30 min washes of 100% acetone. Subsequently, the a-polar lipids were extracted by three incubations in a 60%:40% (*v*/*v*) hexane:acetone solution (8 h each). Then, the defatted tissue was gradually rehydrated and subjected to 0.25% Trypsin–EDTA degradation overnight at room temperature (RT). The tissue was then thoroughly washed with PBS, followed by incubation with 1.5 M NaCl for 24 h (including three solution changes). After 24 h the tissue was washed in 50 mM Tris (pH 8.0), 1% triton-X100 solution for 1 h. The decellularized tissue was washed in PBS followed by double distilled water (DDW) and then frozen (−20 °C) and lyophilized. After lyophilization, the decellularized omentum was grinded into a coarse powder using a Wiley Mini–Mill (Thomas Scientific, Swedesboro, NJ, USA) and then frozen until further use. Dry, milled omentum decellularized ECM (dECM) was enzymatically digested by adding a 1 mg/mL solution of porcine pepsin in 0.1 M HCl. The dECM was digested for 64–72 h at RT under constant stirring until the liquid was homogenous with no visible particles. Subsequently, the salt concentration was adjusted to physiological levels using ×10 phosphate-buffered saline (PBS) and the pH was raised to 7.2–7.4 using 5 M NaOH. Raising the pH terminates pepsin activity. The final concentration of decellularized omentum in the titrated solution was 1 or 1.5% (*w*/*v*).

#### 4.2.2. YIGSR-LF200 Alginate Production

Immobilization of the YIGSR peptide to sodium alginate was carried out utilizing the aqueous carbodiimide chemistry [66] with modifications. Briefly, 1.2 mmol of the activator EDAC was added to 30 mL of sodium alginate dissolved to 1% (*w*/*v*) in 0.1 M MES buffer, pH 6.5. Then, 0.6 mmol of Sulfo-NHS was added to stabilize the reactive EDAC intermediate against competitive hydrolysis, thereby achieving a high efficiency of peptide binding. The NH_2_-GGGGYIGSRGGGG-Me peptide was conjugated to alginate via an amide bond between the terminal amine of the peptide and the carboxylate on alginate. The chemistry was performed using 0.04 mg peptide per 1 mg sodium alginate. The final YIGSR–LF200 alginate product was purified by dialysis (10,000 MWCO) in X0.5 PBS for 3 days and then lyophilized. The dried product was resuspended in 150 mM NaCl solution to achieve a final concentration of 1.8% YIGSR-LF200 (*w*/*v*).

#### 4.2.3. YIGSR-ECM Hydrogel Production

The hybrid YIGSR-ECM hydrogel was achieved by mixing 1.5% ECM hydrogel and 1.8% YIGSR-LF200 solution in a 2:1 ratio (*v*/*v*). The combined solution was stirred for 12 h at 4 °C. The final concentration of the hybrid hydrogel was 1% ECM:0.6% YIGSR-LF200.

#### 4.2.4. YIGSR-ECM Hydrogel Crosslinking

YIGSR-ECM hydrogel samples were crosslinked using both ionic and thermal (physical) crosslinking. First, the samples were ionically crosslinked using calcium by exposing the samples to calcium containing solutions for 60 s and then washing the samples with Hank’s Balanced Salt Solution (HBSS). The calcium crosslinking solutions contained 25 mM calcium D-gluconate supplemented with NaCl to achieve a total physiological osmolarity of 300 mOsm.

#### 4.2.5. Rheological Properties

A volume of 200 μL droplets of 1% ECM hydrogel and the YIGSR-ECM hydrogel were prepared using a high-viscosity pipette and were either incubated at 37 °C in a humidified, 5% CO_2_ incubator for 30 min or ionically crosslinked using calcium, as previously described, and then incubated. Rheological measurements were performed using a Discovery HR-3 Hybrid Rheometer (TA Instruments, New Castle, DE, USA) with 8 mm diameter parallel-plate geometry. The samples were loaded at a temperature of 37 °C, and their storage and loss moduli were measured by performing a frequency sweep between 0.1 and 30 rad/s at a constant 1% strain. At least three gels were assessed and averaged for each condition.

#### 4.2.6. X-ray Photoelectron Spectroscopy (XPS)

XPS measurements for peptide conjugation evaluation were carried out using an ESCALAB QXi XPS spectrometer (Thermo Fisher Scientific). For the analysis, solutions of 1% LF200 sodium alginate and 1.8% YIGSR-LF200 (*w*/*v*) were lyophilized and spread on conductive carbon tape. The samples were analyzed using microfocused radiation with a spot size of 900 μm. A dual-charge compensation system employing electrons and low energy Ar+ ions was employed during the measurements.

#### 4.2.7. Spectrophotometry

Spectrophotometry measurements for peptide conjugation evaluation were carried out using a Spectrophotometry ND-1000 spectrometer (Marshall scientific, Hampton, NH, USA). For the analysis, 3 μL samples of 1% LF200 sodium alginate and 1.8% YIGSR-LF200 (*w*/*v*) solutions were measured for absorbance at 280 nm. At least three samples were measured for each condition. Based on the molar extinction coefficient and Beer–Lambert law, the protein concentration was calculated using the following formula:

(1) $$\;C_{peptide}=\frac{Absorbance_{280nm}\cdot M.W.\;}{\varepsilon_{molar}}$$

where: $C_{peptide}\;$is the concentration of the peptide in mg/mL, ***M.W.*** is the molecular weight of the peptide, $Absorbance_{280nm}\;$ is the difference in the absorbance measured using NanoDrop at 280 nm for the two sample groups and $\varepsilon_{molar}$ is the extinction coefficient of the peptide.

#### 4.2.8. HUVECs Culture

Primary HUVECs were cultured in EGM BulletKit Medium supplemented with additional 3% FBS. The medium was replaced every other day. Cells were passaged by dissociating with 0.25% Trypsin–EDTA solution. In all experiments HUVECs of passages 2–6 were used.

#### 4.2.9. Induced Pluripotent Stem Cell (iPSC) Culture

iPSCs were cultivated on 10 cm culture plates pre-coated with Matrigel^TM^, diluted to 250 μg/mL in DMEM/F12. Cells were maintained in NutriStem^TM^ medium containing 1% Penicillin/Streptomycin and cultured under a humidified atmosphere at 37 °C, 5% CO_2_. Medium was refreshed daily, and cells were passaged weekly by treatment with ReLeSR, followed by mechanical trituration.

#### 4.2.10. EC Differentiation from iPSCs

Cells were differentiated as previously described with modifications [57,67]. Briefly, human iPSCs were dissociated on day 0 with Accutase and replated on Matrigel^TM^, diluted to 50 μg/mL in DMEM/F12, coated plates. Cells were seeded at a density of 47,000 cells/cm^2^ and maintained in NutriStem^TM^ medium containing 1% Penicillin/Streptomycin and 10 μM Y-27632 ROCKi. On day 1, the medium was replaced with mesoderm induction medium containing a 1:1 (*v*/*v*) mix of Neurobasal and DMEM/F12 supplemented with L-Glu, N2 and B27 minus retinoic acid with 25 ng/mL BMP4 and 8 μM CHIR99021. The media was not changed for 3 days to induce a mesoderm state. On day 4, the medium was changed to EC induction medium consisting of StemPro-34 SFM medium supplemented with 200 ng/mL VEGF165 and 2 μM forskolin. The EC induction medium was changed daily. On day 7, the cells were dissociated with Accutase and magnetic-activated cell sorting (MACS) was used to separated for CD31+ CD144+ cells. The sorting was performed using a manual MACS^®^ magnetic separator and magnetic beads conjugated antibodies. The CD31+/CD144+ cells were seeded onto cell culture treated flasks and cultured in EGM-2 supplemented with 20 μM SB431542. Media was replaced every other day. When the cells reached ~90% confluence they were either passaged using 0.25% Trypsin–EDTA solution or cryopreserved.

#### 4.2.11. Flow Cytometry

Cells were dissociated with Accutase, centrifuged at 300 g, and resuspended in flow cytometry staining buffer. The cells were aliquoted for controls and isotype and stained. The antibodies used were: for the iPSCs assay SEA-5 and control antibody, Oct-4 and REA control antibody, and for the iPSCs-ECs assay—CD31 and REA control antibody. Data were collected on a CytoFLEX S Flow Cytometer (Beckman Coulter, Brea, CA, USA) and analyzed using their CytExpert software (Version 2.5.0.77).

#### 4.2.12. The 3D Cell Encapsulation in Hydrogels

To assess self-assembly of the cells in 3D, cells were mixed inside the different hydrogels at a concentration of 10 × 10^6^ cells/mL hydrogel. Next, 3 μL droplets were created from the hydrogel-cell mix. The droplets were then either incubated at 37 °C in a humidified, 5% CO_2_ incubator for 30 min or ionically crosslinked using calcium, as previously described, and then incubated. Upon crosslinking, media was added to the droplets, which were then cultured in a humidified, 5% CO_2_ incubator.

#### 4.2.13. Proliferation Assay

For the proliferation assay, 3D hydrogel-cell droplets were prepared inside 24-well plates as previously described. For each time point, at least three measurements were recorded per group. PrestoBlue™ reagent was added to each well in a 1:9 ratio with cell medium and incubated for 2–3 h, depending on the time until color change was observed on day 0. The absorbance was measured at 570 nm (600 nm serving as the reference wavelength) using an InfiniteM200Pro plate reader (Tecan, Mannedorf, Switzerland). All values were normalized to Day 0.

#### 4.2.14. Viability Assay

For the viability assay, 3D hydrogel-cell droplets were prepared inside 24-well plates as previously described. The viability of cells in the droplets or printed constructs was determined using a live/dead fluorescent staining with 7 μg/mL fluorescein diacetate and 5 μg/mL propidium iodide for 20 min at 37 °C. Cells were visualized by an inverted fluorescence microscope (Nikon Eclipse TI, Tokyo, Japan).

#### 4.2.15. The 2D Cell Seeding on Hydrogels

To adhesion and spreading of the cells in 2D, cells were seeded onto the different hydrogels. First, 100 μL sheets of the different hydrogel were prepared inside 24-well plates. The sheets were then either incubated at 37 °C in a humidified, 5% CO_2_ incubator for 30 min or ionically crosslinked using calcium, as previously described, and then incubated. Next, 0.1 × 10^6^ cells inside 7 μL media were seeded onto the hydrogel sheets. The sheets were then incubated at 37 °C in a humidified, 5% CO_2_ incubator for 30 min to allow the attachment of the cells. After 30 min, media was added to the droplets, which were then cultured in a humidified, 5% CO_2_ incubator.

#### 4.2.16. Immunofluorescence Staining

After the culture medium was removed, the samples were washed twice with PBS. Next, cells were fixated using 3.5% formaldehyde in PBS for 30 min at RT. The samples were then washed 3 times with PBS. The samples were permeabilized and blocked with 0.1% X100 Triton in blocking solution (2% BSA in PBS) for 60 min at RT. Primary antibodies mouse anti-Oct3/4 (1:250), rabbit anti-Ki67 (1:250), rabbit anti-ZO1 (1:200), mouse anti-CD-31 (1:250), mouse anti-CD144 (1:200) were diluted in blocking solution and added to the samples for 1 h at RT or at 4 °C over night. Secondary antibodies goat anti-rabbit Alexa 488 (1:250), goat anti-mouse Alexa 647 (1:250), goat anti-mouse Alexa 555 (1:250) were incubated for 1.5 h. For nuclei detection, Hoechst (1:20) was added along with the secondary antibodies. Phalloidin staining was performed by adding phalloidin conjugated to iFluor 647 (1:1000) or phalloidin conjugated to iFluor 555 (1:1000). Images were taken using confocal microscopy (Nikon Eclipse Ni, Tokyo, Japan) or an inverted fluorescence microscope (Nikon Eclipse TI).

#### 4.2.17. High-Resolution Scanning Electron Microscopy (hrSEM)

Samples for hrSEM were fixed with 2.5% glutaraldehyde 2 h at RT followed by a graded incubation series in ethanol–water solutions (50–100% (*v*/*v*)). Then, the samples were critical point dried, sputter-coated with gold in a Polaron E 5100 coating apparatus (Quorum technologies, Lewis, UK) and observed under GeminiSEM 300 hrSEM (Zeiss, Oberkochen, Germany).

#### 4.2.18. Hydrolytic and Enzymatic Swelling/Degradation Assay

In vitro degradation tests were conducted on 200 μL samples of YIGSR-ECM hydrogels. As described previously, hydrogel samples were crosslinked thermally and ionically. After crosslinking, samples were weighted and transferred to 35 mm plates (four samples per group). Each plate was filled with either PBS (pH 7.4), alginate lyase (1 U/mL) solution or collagenase type 2 (10 μ/mL) solution for hydrolytic or enzymatic degradation studies. Enzyme solutions were prepared in PBS. Samples were incubated at 37 °C and weight measurements were taken at 0, 24, 28, 72 and 96 h for both hydrolytic and enzymatic degradation sets. After each measurement, fresh PBS and enzyme solutions were added to each experimental group. Weight ratios of hydrogels were determined using the following formula:

(2) $$Weigth\;Ration=\frac{Weight\;(t>0)}{Weight\;\left(t=0\right)}$$

#### 4.2.19. Bio-Inks Preparation

For the patch bulk bioink preparation, 1% ECM hydrogel (*w*/*v*) was used. The ECM hydrogel was printed directly (acellularly) or mixed with cells to produce a cell-laden bioink. When printed with cells inside, dissociated iPSC-derived ECs were dispersed in EGM-2 medium and mixed with the ECM hydrogel to achieve a cell concentration of 10 × 10^6^ cells/mL. The bioink was then loaded into a pressure syringe and kept at 4 °C.

For the BM bioink preparation, the YIGSR-ECM hydrogel was used. The YIGSR-ECM hydrogel was printed directly (acellularly). The bioink was then loaded into a gas-tight syringe and kept at 4 °C.

For the sacrificial bioink and support bath, 0.9% (*w*/*v*) xanthan gum (XG) was used. First, 1.11% (*w*/*v*) pure XG was prepared as published before [68]. Briefly, XG was dissolved in DDW supplemented with 150 mM NaCl, to reach a concentration of 4% (*w*/*v*) in the final solution. Following autoclaving for sterilization, the preparation was supplemented with 5 M NaOH solution and mixed thoroughly to achieve a homogenous mixture containing 1 M NaOH. After 24 h of incubation for at RT, the mixture was poured to a large sterile syringe and extruded into a perforated vessel. This vessel was soaked in a solution of 50% (*v*/*v*) ethanol that is 10-foldlarger in volume. In this manner, the XG remained insoluble while the ions from the NaCl and NaOH diffuse into the surrounding liquid. The 50% ethanol solution was changed daily until pH reached neutrilization. The solution was replaced with a 70% (*v*/*v*) ethanol solution. When the pH of the solution remained stable between solution exchanges, the XG was removed from the solution and dried in under airflow.

Next, salts were added to the 1.11% (*w*/*v*) pure XG to achieve a final concentration of 0.9% (*w*/*v*) XG with 150 mM NaCl and 10 mM calcium D-gluconate.

The sacrificial cell-laden XG bioink was generated by mixing dissociated iPSC-derived ECs dispersed in EGM-2 medium with the salinized 0.9% (*w*/*v*) XG to achieve a cell concentration of 40 × 10^6^ cells/mL. The bioink was then loaded into a gas-tight syringe (Hamilton) and kept at 4 °C.

#### 4.2.20. Vascular Patch Printing Process

Vascular patches were printed using a 3D discovery Evolution^®^ printer (RegenHu, Villaz-Saint-Pierre, Switzerland). The bioinks were extruded through different gauge needles (25 G for the bulk and BM bioinks, 30 G for the sacrificial bioink) onto 12 wells filled with a support bath made of XG. First, ECM hydrogel based bulk bioink was extruded in a crisscross geometry, creating the six lower layers of the patch. Embedded within the sixth layer, the BM bioink was deposited to generate the vascular network. Next, the sacrificial bioink was deposited into the vascular pattern previously created by the BM bioink using a smaller needle gauge. On top, another six layers of crisscrossed ECM hydrogel based bulk bioink were extruded, encapsulating the printed vessels. The printed patches were then incubated at 37 °C for 30 min to cure the ECM hydrogel, followed by submerging in EGM-2 media for XG dissolvement. Patches were cultured in a defined media as previously described [69] with the addition of VEGF in the final concentration of 0.1 μg/mL.

#### 4.2.21. Statistical Analysis

Statistical analysis data are presented as the means ± standard deviation. Differences between samples were assessed by Student’s *t*-test where *p* < 0.05 was considered significant. Analyses were performed using GraphPad Prism 9.0 for Mac (GraphPad Software, version 10.0.2). Images were analyzed using ImageJ (NIH, version 1.5.3).

## Data Availability

Not applicable.
